# Evidence for Vertical Transmission of HPV from Mothers to Infants

**DOI:** 10.1155/2010/326369

**Published:** 2010-03-14

**Authors:** Elaine M. Smith, Michael A. Parker, Linda M. Rubenstein, Thomas H. Haugen, Eva Hamsikova, Lubomir P. Turek

**Affiliations:** ^1^Department of Epidemiology, College of Public Health, University of Iowa, Iowa City, IA 52242, USA; ^2^Department of Obstetrics/Gynecology, College of Medicine, University of Iowa, Iowa City, IA 52242, USA; ^3^Veterans Affairs Medical Center, Iowa City, IA 52240, USA; ^4^Department of Experimental Virology, Institute of Hematology and Blood Transfusion, U nemocnice 1, 128 20 Prague 2, Czech Republic

## Abstract

Few large studies have evaluated concordance based on a broad spectrum of human papillomavirus (HPV) types in oral and genital specimens of mothers and their recently born infants. This information is important in determining whether HPV vaccines administered prior to pregnancy may be useful for preventing vertical transmission. HPV DNA was positive in 30% of mothers and 1.5% of newborns. Maternal/newborn concordance (HPV+/+ or HPV−/−) was 71%. Among HPV DNA+ mothers, only 3% of their infants were DNA+ and only 1 pair had the same HPV type. Among HPV− women, 0.8% of infants were HPV+. HPV DNA detected in hospitalized newborns reflects current infection transmitted to infants during pregnancy or delivery. None of the mother/baby HPV DNA+ concordance pairs detected viral types found in HPV vaccines suggesting that vaccination prior to pregnancy is unlikely to be efficacious in preventing vertical transmission.

## 1. Introduction

Human papillomavirus (HPV) is well recognized for causing laryngeal papillomatosis, genital warts, and cancer [1, 2]. HPV types are classified as low-risk, nononcogenic, types, associated with anogenital warts and laryngeal papillomatosis or high-risk, oncogenic, HPV types associated with cancers of the cervix, anogenital areas, and head and neck [3]. The most prevalent HPV types associated with genital and oral cancers are HPV-16, 18, and 33. HPV-6 and -11 are most commonly associated with neonatal laryngeal papillomatosis and genital warts.

Although the predominant mode of viral transmission occurs through sexual contact, HPV also has been found in virginal women prior to first coitus [1, 2, 4]. Studies suggest that the virus can be transmitted from mother to infant before or during childbirth [5–11]. We and others have found that the risk of vertical transmission of HPV DNA to the oral or genital mucosa of newborns to be rare, 1–5% [6, 10, 12, 13]. In contrast, other studies suggest the vertical transmission is common, 40%–80% [7, 12, 14]. Several studies of persistent HPV DNA, a method for distinguishing inoculation from true infection, reported maternal/newborn concordance after birth to be maintained between 37%–83% at 6 weeks to 6 months after birth [15, 16] whereas another study has shown a lower 10% prevalence in infants at 24 months of follow-up [14]. Maternal HPV positivity is consistently a risk factor for HPV infection in infants [14–16]. The prevalence of this nonsexual mode of viral transmission may have an important impact on vaccination strategies and clinical management of infected women in family planning before pregnancy. Thus it is important to not only clarify the frequency of transmission and concordance but also to determine whether the same HPV types are detected in mother and infant in an environment controlled for other potential sources of HPV transmission. The purpose of this study was to assess maternal risk factors for transmission of HPV to their newborns prior to hospital discharge and to assess the level of HPV type specific concordance based on maternal/infant antibodies and cytologic DNA from genital and oral specimens.

## 2. Methods

### 2.1. Participants

Between 1997 and 2000, all pregnant women, ages 18 and over, who were being seen in their third trimester of pregnancy during routine obstetric examinations (*n* = 582) were recruited into the study at the University of Iowa Hospitals and Clinics, Department of Obstetrics/Gynecology. The study included only healthy women with normal pregnancies. Mothers were excluded if they were having complicated pregnancies, had a language barrier, were mentally unable to consent, or were not going to deliver at the research hospital site and thus would not be eligible for the aims of the study. All participants signed an approved Human Subjects Consent form. Not all women who were recruited could be included in the final analyses because some women delivered elsewhere or the cord blood was not available at delivery. Among 333 included pregnant women who were included in the study analyses, HPV results were evaluated by obtaining cervical and oral cell specimens and by collecting peripartum serology. Samples from the 333 newborns were obtained from oral and genital areas and from cord blood to test for HPV status. There were 193 male and 140 female newborns in the study. No same sex couples or nonbiologic partners were identified during the study recruitment.

### 2.2. Data Collection

After a university-approved human subject consent form was signed by the mothers, they completed a self-administered questionnaire of information about demographics, reproductive, sexual history, and HPV-related lesions. Newborns were evaluated by the attending pediatrician for gestational age at birth, gender, type of delivery, bottle/breast feeding preferences, medical problems at birth, and number of hours after delivery when the HPV specimen was obtained from the infant. Maternal cervical swabs and oral rinses were obtained during the third trimester of pregnancy and immediately before delivery by trained obstetricians and nurse practitioners. Maternal serum samples were obtained aseptically during the delivery process. Prior to discharge, infants were tested for HPV DNA by swabbing their genital and oral regions. The procedures have been described previously [15]. Infant oral and genital specimens were taken at a median of 42 hours after birth to reduce the risk of viral inoculation but not infection of the tissue. The minimum time after birth before specimens were collected to evaluate HPV DNA was set at 24 hours; however, because two women were planning to be discharged prior to that time, specimens in their newborns were collected <24 hours after birth. In males that were circumcised a portion of tissue was tested for HPV DNA. Infant serum samples were obtained from the placenta at birth.

### 2.3. Laboratory Methods

Serological testing was performed to determine if an individual had antibodies to HPV-specific capsid proteins (virus-like particles/VLPs). The procedures have been described elsewhere [17–21]. The presence of antibodies to antigens derived from HPV-specific proteins was determined using an ELISA test using HPV types 16, 18, 31, and 33 VLPs as antigens [22] and these were defined as HPV high-risk types (HPV-HR). HPV 6 and 11 VLPs, defined as low-risk types (HPV-LR) were not available at the time these analyses were tested. Background reactivity was determined in wells without antigen. Their absorbances were subtracted from corresponding values obtained in the presence of antigen. Control sera known to be positive and negative were tested on each plate. For the cutoff level calculation, above which optical density values were considered positive, 10 standard sera from regular blood donors known to be HPV-antibody negative were included on each plate. The means and SD were calculated for each antigen separately, the cutoff point was represented as the mean absorbance of the control specimen plus 3 SD. 

Sample preparation, PCR analyses, DNA hybridization, and HPV typing procedures for assessment of exfoliated oral cells were based on a standard protocol described previously [23, 24]. Each PCR reaction included primers to amplify the *β*-globin gene [25] to verify adequate DNA and adequacy of the PCR amplifications of the cellular DNA. All oral cell specimens tested positive for the *β*-globin gene. Two percent of the DNA (typically 50–200 ng) extracted from cell specimens was PCR-amplified with MY09 and MY11 primers [26] to detect HPV and a primer (HMB01) designed to amplify HPV-51 to improve detection of this type [27]. The PCR product first was examined for the presence of a 400 nt HPV amplified band on ethidium bromide stained 1% agarose gels. An aliquot of the PCR product of all those specimens that were HPV-negative after staining was hybridized by the dot blot method with ^32^P-labeled probes for a more sensitive detection of HPV DNA. Those samples positive only after the membrane hybridization underwent hemi-nested PCR-amplification with MY09 and GP5+ primers [28]. DNA sequencing was used to determine the HPV types in each HPV-positive specimen, performed by dye-termination on a DNA sequencer (Applied Biosystems-PE). The nucleotide sequences were compared to GenBank sequences using the BLAST program [29].

### 2.4. Statistical Analysis

Logistic regression was used to estimate odds ratios (ORs) and 95% confidence intervals (CIs) for HPV seropositivity associated with mother and newborn characteristics adjusting for number of sexual partners and maternal history of an HPV-related disease. Concordance was examined using McNemar's test for matched pairs in 2 by 2 tables utilizing mother and newborn VLP and HPV DNA results. The Kappa statistic was used to measure agreement in HPV positivity status between mother and newborn sera samples. Statistical significance was noted at  .05 and all tests were 2-sided. All analyses were performed using the SAS statistical package [30].

## 3. Results

Table 1 shows the demographic characteristics and risk factors for HPV DNA in 333 healthy pregnant women positive for one or more HPV types detected in the cervix and/or oral mucosa. The mean age at delivery was 29 (range: 18–44 years), 16% were nonwhite/nonHispanic races, and 77% had greater than high school education. More than three quarters had sexual intercourse before the age of 21, two-thirds had 3 or more partners before the current pregnancy, over half had two or fewer prior pregnancies, and most had used oral contraceptives. Over 25% of the women had a history of an HPV-related lesion and five had had histologically-confirmed cervical cancer. The majority of newborns were males (58%), ≥39 weeks gestation (67%), and born by vaginal delivery (89%). 

Risk factors included in Table 1 are based on HPV DNA since it is indicative of current infection as opposed to HPV VLPs which represent both past and current infection. After adjustment for number of partners and history of an HPV-related disease in mothers, younger age, lower education, greater number of partners, smoking, cervical dysplasia, and cervical cancer were associated with an increased risk of maternal HPV DNA. Newborn HPV DNA positivity was not associated with gender, feeding method, type of delivery, or gestational age (data not shown). Among the 333 women, there were 295 (87%) vaginal deliveries and 38 (13%) C-sections. Among the 99 DNA positive mothers, the proportion of vaginal (*n* = 86) and C-section (*n* = 13) deliveries were also 87% and 13%, respectively. For HPV DNA transmission, only 3 (1%) of the 86 vaginal deliveries were concordant and none of the 13 DNA positive C-sections (*P*-value > .99). For serologic transmission, 35% (30/86) of the vaginal deliveries and 38% (5/13) of the C-sections were concordant. After adjusting for a history of HPV disease, the OR comparing vaginal to C-section deliveries was 1.1 (0.1–23.1) for DNA transmission and 0.9 (0.3–3.0) for serologic transmission. There were no significant associations between seropositivity to anti-HPV VLPs (HPV-16, 18, 31, 33) in mothers and risk of HPV-HR DNA for these same four HPV types (data not shown).

HPV frequency and types in mothers and newborns are shown in Table 2. HPV DNA, which tests for a current infection, showed that among mothers, 30% were HPV DNA positive: 28% in the cervix and 3% in the oral mucosa; and 22% of mothers had an HPV-HR type. Multiple types were detected in 13.5% (*n* = 45), only in the cervix. The two most common HPV-HR types in mothers were HPV-16 (33%) and HPV-31 (29%) and these types were more frequent in both cervical and oral mucosa. Less common HPV-HR DNA types included 18, 33, 39, 51, 56, 58, 59, 66, and 70. The overall prevalence of HPV-16, 18, 31, and 33 DNA was 16% in mothers. The overall prevalence of HPV-LR DNA in mothers was 8% and the most common LR types were HPV-53 (19%), HPV-61 (14%), and HPV-83 (14%); other less frequently detected types were HPV-6, 11, 54, 69, 84, and several unnamed types. The table also shows the frequency of HPV-6, 11, 16, 18 types which are found in current HPV vaccines. Maternal and newborn data indicated that only about one-third of their HPV+ DNA infections included these four vaccine types. The prevalence of HPV DNA in newborns was 1.5% (Table 2) and there was no significant difference in sample collection time between the HPV+ and HPV− newborns: 40 hrs. versus 41.5 hrs (*P* > .10). Three newborns were detected with HPV DNA in oral samples: 2 HPV-HR types (HPV-16 and 51) and 1 HPV-LR type (HPV-61). Two newborn HPV-LR vulvar samples also were positive for HPV-61 whereas none of the circumcision tissue samples was positive. No multiple infections were found in the HPV DNA of newborns.

The seropositivity rate to VLPs 16, 18, 31, and 33 was 32% in mothers and 31% in newborns. The prevalence was similar in mother and infant for each VLP type tested (Table 2). Among the seropositive mothers, 58% were detected with a single infection and 42% with multiple infections. In infants, 44% were seropositive for multiple types. In mothers, 13% were HPV DNA+/VLP+, 17% were DNA+/VLP−, 20% were DNA−/VLP+, and 50% were DNA−/VLP−. In the DNA+/VLP+ mothers, the type-specific concordance ranged from 25%–50%, thus a high percentage (50–74%) of DNA+ for HPV-16, 18, 31, or 33 were type-specific seronegative. None of the infant DNA+ types matched their anti-VLP antibody types.

The mean HPV specimen collection interval between enrollment at the third trimester and delivery was 71.4 days. There was no significant association between the interval between the third trimester and delivery specimen collection period and subsequent risk of HPV transmission. Based on serology, the mean intervals between HPV seronegative versus HPV seropositive transmission was 74 days and 60 days, respectively, (adjusted *P*-value = .20). Based on DNA transmission, the mean intervals for HPV DNA-negative versus HPV DNA-positive transmission was 63 days and 49 days, respectively (adjusted *P*-value = .59). The overall mean interval time (based on both serology and DNA) was not significantly related to the transmission rate. 

Next we examined concordance between maternal and newborn samples for anti-HPV VLPs (Table 3). There was a high concordance (93%) and low discordance rate between maternal and newborn antibodies (*P* = .30) and high agreement in HPV seropositivity (*K* = 0.84, 0.78–0.90). Type specific concordance rates were between 96%–97%.

The HPV DNA+ concordance rate between mothers and newborns was 71% (*P* < .0001), mostly due to HPV– findings in both groups (Table 4). If the mother was HPV+, the risk in the infant was increased (OR  =  3.6, CI: 0.6–22.0) but the low number of positive newborns resulted in the wide CIs. Among the 30% HPV+ mothers, 3% (*n* = 3) of their infants were HPV+ compared to 0.8% (*n* = 2) of infants in HPV– mothers. Although there were three mother/baby pairs that were both HPV+, HPV type-specific concordance occurred in only one case, for HPV-51 in the maternal cervix and oral mucosa of the newborn. The other two HPV+ mother/baby pairs were HPV-39/HPV-61 and HPV-99/HPV-61. The two HPV+ infants with HPV-mothers were detected with HPV-16 and HPV-61. Only one of the five DNA+ newborns had a type contained in current HPV vaccines (HPV-16). Thus the findings were substantially different from those of serology which were essentially those of the mother whereas the DNA findings represented current infection in both mother and infant.

## 4. Discussion

This large epidemiologic study evaluated HPV concordance between mothers and newborns by examining viral frequency and types both in serum and in cytology and identified risk factors for vertical transmission. Previous studies [6, 7, 14], including ours [10, 11], have evaluated HPV vertical transmission by examining the prevalence of HPV DNA positivity in mothers and newborns, and less often, in investigations with sufficient DNA of oral/genital specimens [6, 13, 15, 16] have determined HPV types and type-specific concordance between mother and newborn. In past studies, we found that the maternal prevalence rate of HPV DNA from oral/genital specimens was 31% whereas it was <2% in her infant [10, 11] rates similar in this investigation based on a different study population. Although the seropositivity rates for HPV-16, 18, 31, 33 in this analysis were similar to the maternal DNA+ rates overall, 31–32%, only half of the HPV DNA+ mothers were detected with these same four HPV serologic high-risk types; and the single HPV-16 DNA+ infant was HPV seronegative. Relevant to this finding is the fact that there was no association between type of delivery and rate of vertical transmission; thus vaginal delivery does not appear to convey a greater risk of HPV from mother to newborn even where there was concordance in HPV type.

Analyses of HPV serologic concordance in mothers and newborns have not been performed previously primarily because mothers provide immunologic antibodies for their newborns in the first 3–6 months of life and there is likely to be little discordance in HPV status or types between mother and newborn. This was indeed the outcome with few exceptions, most likely due to either maternal viral clearance when she tested seronegative and her newborn tested seropositive or when infants had not yet mounted a sufficient antibody level from mothers who recently acquired an HPV infection and tested seropositive. Thus, this is the first study to have validated fetal acquisition of maternal IgG.

Since a comparison of maternal anti-HPV serologic findings does not provide a valid measure of transmission of infection to infants, the clinical focus is limited to concordance in DNA types from exfoliated cells. This source of HPV detection has the advantage of measuring current infection in the individual unlike serologic markers. Supported by our former [10, 11] and current investigation, and those of others with large (*N* > 150) maternal/newborn samples [6, 13], vertical transmission of HPV DNA detected in genital and oral cells is uncommon, < 5%. Despite that low prevalence, we found that the risk of vertical infection was increased if the mother was detected with HPV prior to delivery or had an HPV-related lesion prior to delivery (OR = 3.0, 1.8–4.0). Mothers with a history of an HPV-related disease had a transmission rate of 47% versus 23% in women without a history (*P* < .0001). Yet only one of the five HPV+ newborns was detected with an HPV vaccine type (HPV-16). Furthermore, in this study the genitals in newborns were as likely to be infected as the oral mucosa; thus concern that vertical transmission of HPV will primarily affect the oral mucosa (and thus the potential for laryngeal papillomatosis) does not attend to the larger concern about transmission to genital mucosa areas. The risk of genital dysplasia and cancer in adolescents and young adults based on vertical transmission may be important, as it is for HPV-related oral papillomatosis.

Although others suggest that the frequency of vertical transmission is much higher [14–16], these findings are most likely due to small sample sizes that produce unstable frequency rates, and inoculation rather than infection from specimens taken immediately after birth especially those from nasopharyngeal aspirates, or PCR contamination. We prepared and maintained collected specimens, and performed PCR preparation in a separate building from that where the PCR assay was conducted to reduce contamination. In addition, we examined the most commonly detected HPV types by sequencing and verified that there was not a high frequency of any one variant and that the variant in mother/baby was the same. 

We are among one of the few to have examined HPV DNA type-specific maternal/newborn concordance in a large patient population [10–13]. Among mother/baby pairs infected with HPV, we found low type-specific concordance, 17% (1/6), in a study of 574 mother/baby pairs and 20% (*n* = 1/5) in this analysis, both studies using DNA sequencing to identify a wide variety of types. In contrast, Tseng et al [12], detected 100% of HPV-16/18 concordance in oral/genital specimens of 301 mother/baby pairs (27/27) as did Tenti [13] (11/11: 4 HPV-16/18, 7 other unspecified types matched), but the latter study finding was based on newborn nasopharyngeal aspirates taken at birth and likely reflects maternal contamination to the newborn. These two studies limited assessment to HPV-16 and -18 which accounted for only half of the HPV DNA types detected. Thus the frequency of vertical transmission in type-specific concordance needs further investigation.

It was surprising that the most common HPV DNA types that have been reported in the oral mucosa, HPV-16, 18, 31, and 33, and which are among the most frequent identified in the cervix [31], did not have type-specific concordance to VLP types in the same individual, mother or newborn. We found that 20% of DNA negative mothers were VLP seropositive for one of the four HPV types. Possible reasons for this discordant finding may be due to inadequate HPV copy number to detect infection (latent infection), clearance of infection prior treatment of an HPV-lesion which may have eliminated the lesion with the infection, or lack of an immune response. A number of large studies of HPV infection in healthy young women have found comparable DNA [32–34] and VLP seroprevalence rates in young women to those in our low-risk pregnant group [35, 36]. Interestingly, there was a high proportion of women (17%) who were DNA positive to HPV-16, 18, 31, or 33 but who did not have anti-VLP antibodies to those specific types. These infections may be associated with the inability of the individual to mount an antibody response, inadequate time for a recent infection to produce an immune response, or low level viral replication that does not produce a detectable antibody response, a finding reported by Nonnenmacher and Schiller as well [36].

Among the strengths of this study was the ability to evaluate type-specific concordance of DNA and serologic HPV results between mothers and newborns. In comparison with other studies which tested for less than six HPV DNA types, our study sequenced HPV DNA and thus tested for all types. The conclusion remains, however, that DNA concordance and thus vertical transmission is low between mother/newborn oral and genital sites. The large sample size in this study allowed investigating the concordance of HPV positivity in mothers and their newborns. The size also allowed not only an assessment of stable prevalence rates but also examination of maternal risk factors for HPV infection in the newborn as a potential method for identifying infants at high-risk of acquiring this viral infection. Based on the low HPV DNA positive maternal/neonate concordance, this suggests that prophylactic HPV vaccination during family planning prior to pregnancy is unlikely to be efficacious in preventing vertical transmission. More importantly, the findings suggest that because vertical transmission of HPV is low, the suggestion that women be vaccinated prior to pregnancy is unlikely to be efficacious.

## Figures and Tables

**Table 1:** Demographic characteristics and risk factors for HPV DNA positivity among mothers (*n * = 333).

Characteristic	*N* (% HPV+)^1,2^	Adjusted OR	
		95% CI^3^	
Age			
≤24	39/81 (48.1)	4.2	(2.2–7.8)
25–29	42/110 (29.1)	2.1	(1.1–3.8)
≥30	28/142 (19.7)	reference	—
Race			
White	83/281 (29.5)	reference	—
Other	16/52 (30.8)	1.2	(0.6–2.4)
Education			
≤12	37/78 (47.4)	3.4	(1.8–6.7)
13–16	39/142 (27.5)	1.3	(0.7–2.5)
≥17	23/113 (20.4)	reference	—
Age first intercourse			
<20	88/258 (34.1)	1.9	(0.9–4.1)
≥20	11/75 (14.7)	reference	—
Number of partners			
1-2	15/105 (14.3)	reference	—
≥3	83/225 (36.9)	3.1	(1.7–5.8)
Parity			
1-2	56/193 (29.0)	reference	—
≥3	43/140 (30.7)	1.0	(0.6–1.6)
OC Use			
Never	16/44 (36.4)	reference	—
Ever	83/289 (28.7)	0.5	(0.2–1.02)
Smoking			
Never	51/220 (23.2)	reference	—
Ever	48/113 (42.5)	1.8	(1.04–3.0)
Alcohol			
Never	66/210 (31.4)	reference	—
Ever	33/123 (26.8)	0.6	(0.3–0.96)
HPV-related disease			
Never	62/244 (25.4)	reference	—
Genital warts	15/39 (38.5)	1.4	(0.7–2.8)
Cervical dysplasia	30/71 (42.3)	1.8	(0.98–3.1)
Cervical cancer	4/5 (80.0)	8.0	(0.9–73.4)
Pap smear			
WNL	57/219 (26.0)	reference	—
ASCUS	12/42 (28.6)	0.8	(0.4–1.8)
L-SIL	30/71 (42.3)	1.1	(0.4–3.3)
HPV VLP			
HPV–	57/225 (25.3)	reference	—
HPV+^4^	42/108 (38.9)	1.3	(0.8–2.2)

^1^HPV DNA types include HPV-6, 11, 16, 18, 31, 33, 39, 51, 53, 54, 56, 58, 59, 61, 66, 69, 70, 83, 84, other/unnamed types; ^2^Based on HPV+ in cervical/genital and/or oral samples; ^3^adjusted for number of sexual partners and history of an HPV-related disease; ^4^HPV-HR VLP types HPV-16, 18, 31, 33.

**Table 2:** HPV types and frequencies in mothers and newborns (*N* = 333).

	Mother				Newborn			

	DNA			Serum^3^	DNA			Serum^3^
	Cervical	Oral^5^	Cervical/Oral		Genital	Oral	Genital/Oral	
HPV status	*N* (%)	*N* (%)	*N* (%)	*N* (%)	*N* (%)	*N* (%)	*N* (%)	*N* (%)

HPV+^1^	93 (27.9)	10 (3.0)	99 (29.7)	108 (32.4)	2 (0.6)	3 (0.9)	5 (1.5)	103 (30.9)
HPV-LR^1,2^	27 (8.1)	3 (0.9)	26 (7.8)		2 (0.6)	1 (0.3)	3 (0.9)	0 (0.0)
6	3 (0.9)	1 (0.3)	4 (1.2)	0 (0.0)			0 (0.0)	
11	2 (0.6)		2 (0.6)	0 (0.0)			0 (0.0)	
HPV-HR^1,2^	66 (19.8)	7 (2.1)	73 (21.9)	108 (32.4)		2 (0.6)	2 (0.6)	103 (30.9)
16	22 (6.6)	2 (0.6)	24 (7.2)	47 (14.1)		1 (0.3)		49 (14.7)
18	8 (2.4)		8 (2.4)	46 (13.8)				45 (13.5)
31	19 (5.7)	2 (0.6)	21 (6.3)	41 (12.3)				39 (11.7)
33	2 (0.6)	1 (0.3)	3 (0.9)	40 (12.0)^4^				36 (10.8)^4^
HPV-6,11,16,18	35 (10.5)	2 (0.6)	37 (11.1)		0 (0.0)	1 (0.3)	1 (0.3)	

^1^Percentages based on number of cases; ^2^HPV-HR = high risk types detected (HPV-16, 18, 31, 33, 39, 51, 56, 58, 59, 66, 70); HPV-LR = low risk types detected (HPV-6, 11, 53, 54, 61, 69, 83, 84, other/unnamed types); the number of HR and LR types do not sum to total number of HPV+ in cervical group because results represent the 2 cervical samples combined; and individuals may have multiple types, including both HR and LR types; ^3^number of HR types do not sum to total number of HPV+ because of multiple infections; ^4^data not available, some VLP types not tested.

**Table 3:** Concordance between mother and newborn for anti-HPV VLP 16, 18, 31, or 33 (*N * = 333).

Mother	Newborn	
Antibodies	VLP+	VLP –
	*N* (%)	*N* (%)

VLP +	94 (28.2)	14 (4.2)
VLP −	9 (2.7)	216 (64.9)

**Table 4:** Concordance between mother and newborn for HPV DNA^1^ (*N * = 333).

Mother	Newborn	
DNA^1^	HPV+	HPV –
	*N* (%)	*N* (%)

HPV+	3 (0.9)	96 (28.8)
HPV −	2 (0.6)	232 (69.7)

^1^HPV+ cervical/genital and/or oral for any HPV type.
